# Detection of HBV Covalently Closed Circular DNA

**DOI:** 10.3390/v9060139

**Published:** 2017-06-06

**Authors:** Xiaoling Li, Jinghua Zhao, Quan Yuan, Ningshao Xia

**Affiliations:** 1 State Key Laboratory of Molecular Vaccinology and Molecular Diagnostics, School of Public Health, Xiamen University, Xiamen 361102, China; 15711511022@163.com (X.L.); nsxia@xmu.edu.cn (N.X.); 2 National Institute of Diagnostics and Vaccine Development in Infectious Diseases, School of Life Sciences, Xiamen University, Xiamen 361102, China; 21620111152491@stu.xmu.edu.cn

**Keywords:** detection, hepatitis B virus, cccDNA

## Abstract

Chronic hepatitis B virus (HBV) infection affects approximately 240 million people worldwide and remains a serious public health concern because its complete cure is impossible with current treatments. Covalently closed circular DNA (cccDNA) in the nucleus of infected cells cannot be eliminated by present therapeutics and may result in persistence and relapse. Drug development targeting cccDNA formation and maintenance is hindered by the lack of efficient cccDNA models and reliable cccDNA detection methods. Southern blotting is regarded as the gold standard for quantitative cccDNA detection, but it is complicated and not suitable for high-throughput drug screening, so more sensitive and simple methods, including polymerase chain reaction (PCR)-based methods, Invader assays, in situ hybridization and surrogates, have been developed for cccDNA detection. However, most methods are not reliable enough, and there are no unified standards for these approaches. This review will summarize available methods for cccDNA detection. It is hoped that more robust methods for cccDNA monitoring will be developed and that standard operation procedures for routine cccDNA detection in scientific research and clinical monitoring will be established.

## 1. Introduction

Chronic hepatitis B virus (HBV) infection increases the risk of developing liver fibrosis, cirrhosis and hepatocellular carcinoma (HCC), and it is a leading cause of mortality around the world. There are approximately 240 million chronic hepatitis B (CHB) patients worldwide and 680,000 deaths due to CHB annually [1,2,3,4,5]. Drugs for CHB treatment include interferons—standard and pegylated interferon (PEG-IFN)—and six clinically-approved nucleos(t)ide analogues (lamivudine, adefovir, entecavir, telbivudine, tenofovir and emtricitabine), which have been shown to suppress virus replication, delay the progression of cirrhosis and reduce the incidence of HCC [6,7,8,9], but their curative efficacy is limited because HBV replication resumes after the treatment is stopped. Moreover, interferons have serious side effects, and nucleos(t)ide analogues require long-term treatment for persistent virus suppression [1,2,4,10]. Current therapy rarely achieves a functional cure—Hepatitis B surface antigen (HBsAg) loss or covalently closed circular DNA (cccDNA) inactivation—, let alone complete cure (cccDNA elimination). Even if HBsAg loss is achieved, relapse may occur upon immunosuppression. The key obstacle is the persistence of a HBV replication intermediate known as cccDNA, which exists in the nucleus of infected cell as a plasmid-like molecule and serves as the template for viral RNAs. To the best of our knowledge, the eradication of CHB requires the elimination of cccDNA [3,10,11,12,13,14,15,16,17,18,19].

Despite the pivotal role of cccDNA in the chronicity and durability of HBV infection, little is known about the mechanisms of cccDNA formation, fluctuation and degradation [3,12,16,18]. Research is hindered by certain key obstacles: first, the availability of samples before, during and after antiviral therapy is limited. Second, cccDNA levels are low or even undetectable in patients’ liver tissues and in most current in vitro and in vivo HBV infection models. Finally, and most importantly, there is a lack of a practical, sensitive and accurate method to monitor cccDNA [16,20]. The widely accepted method for cccDNA detection is Southern blotting, which is insensitive, complex, time-consuming and not suitable for high-throughput drug screening. To resolve this problem and facilitate research on cccDNA, many new methodologies, including polymerase chain reaction (PCR)-based methods, Invader assays, in situ hybridization, and surrogates, have recently been applied to detect and quantify cccDNA [20,21,22,23,24,25,26,27,28,29,30,31,32,33,34,35,36,37,38,39,40,41,42,43,44,45,46]. In this review, we summarize and compare currently available approaches for cccDNA detection.

## 2. Formation and Structure of cccDNA

**Figure 1:**
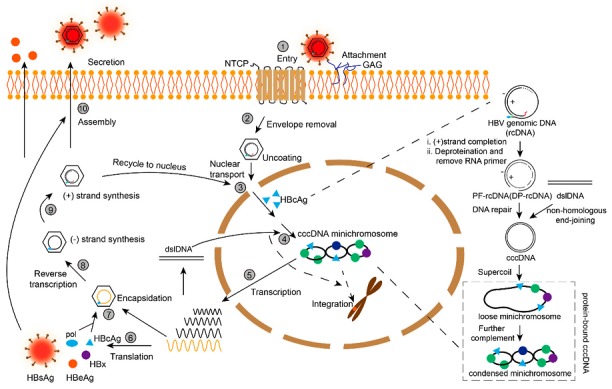
Formation of covalently closed circular DNA (cccDNA) in the hepatitis B virus (HBV) life cycle. ① Attachment and entry of HBV via glycosaminoglycan (GAG) and sodium taurocholate cotransporting polypeptide (NTCP); ② Removal of the envelope and the inner nucleocapsid is released into the cytoplasm; ③ Transport of nucleocapsid into the nucleus pore and release of the polymerase (pol)-linked relaxed circular DNA (rcDNA); ④ Conversion of cccDNA from rcDNA and double-stranded linear DNA (dslDNA). rcDNA is the main source of cccDNA; the incoming or intracellular amplified rcDNA fills in the gap in plus strand and removes the pol and RNA primer from the 5′-terminus of minus and plus strand to form the protein-free (PF)-rcDNA, which is also called deproteinized-rcDNA (DP-rcDNA). The end of both strands of PF-rcDNA is further ligated to form cccDNA. In addition, a fraction of cccDNA is synthesized from dslDNA by non-homologous end-joining (NHEJ). The episomal cccDNA once formed is assembled into minichromosomes including loose or spread and condensed forms bound to histone and non-histone proteins; ⑤ Transcription of viral mRNAs (3.5 kb pregenomic RNA (pgRNA) and precoreRNA, 2.4 kb and 2.1 kb preS/S mRNAs and 0.7 kb X mRNA ) from cccDNA minichromosomes; ⑥ Translation of HBV proteins: hepatitis B surface antigen (HBsAg), hepatitis B e antigen (HBeAg), hepatitis B core antigen (HBcAg), HBV X protein (HBx) and polymerase (pol); ⑦ Encapsidation involving pgRNA, pol and HBcAg; ⑧ Synthesis of HBV minus strand via reverse transcription of pgRNA; ⑨ HBV plus strand synthesis; ⑩ Assembly of capsid containing rcDNA with HBsAg to form virion which secrets out of the cell. Dashed line: Variable 3′-end of the plus-strand; Red line: RNA primer at the 5′-end of the plus-strand; Green line: Redundant sequences at 5′- and 3′-end of the minus-strand; Blue oval: Polymerase.

HBV infection of hepatocytes initiates with concentrated attachment of the HBV virion to cell surface glycosaminoglycans (GAGs) followed by a high-affinity binding of the myristoylated N-terminal PreS1 of the HBV large envelope protein to the sodium taurocholate co-transporting polypeptide (NTCP) receptor on the hepatocytes [47,48]. Following virion entry into the cell (likely by endocytosis), the envelope is removed, and the HBV nucleocapsid releases the enclosed relaxed circular DNA (rcDNA), which is transported to the nucleus. In the nucleus, rcDNA is converted into cccDNA in several steps: the completion of plus-strand DNA synthesis, the removal of the polymerase and RNA primer from minus- and plus-strand DNA, respectively, the ligation of both DNA strand extremities, and incorporation in the nucleosome to form a non-integrated minichromosome [12,49,50,51,52,53,54]. The cccDNA then acts as a template for the transcription of all viral ribonucleic acid (RNA) molecules, including the four major messenger RNAs (mRNAs) encoding the viral proteins: hepatitis B core antigen (HBcAg), hepatitis B e antigen (HBeAg), HBV polymerase (pol), hepatitis B surface antigen (HBsAg) and HBV X protein (HBx). HBV replicates its genomic rcDNA via reverse transcription of pregenomic RNA (pgRNA), one of the four RNA forms, and the resulting rcDNA in the nucleocapsid can be further enveloped and secreted or recycled to the nucleus to amplify the cccDNA pool [55,56,57,58]. In addition to the rcDNA from incoming virions and the intracellular amplification pathway, cccDNA can be generated from double-stranded linear DNA (dslDNA) which is the major precursor for HBV integration by non-homologous recombination; In this process, the generated cccDNA is generally defective due to a loss of sequence fragments [59,60,61]. On the whole, HBV cccDNA has been demonstrated to be organized into nucleosome-decorated minichromosomes consisting of two forms: loose form (or spread form) and condensed form, both bound to histone and non-histone proteins, as shown in Figure 1 [14,50,51,54,57,59,62].

**Figure 2:**
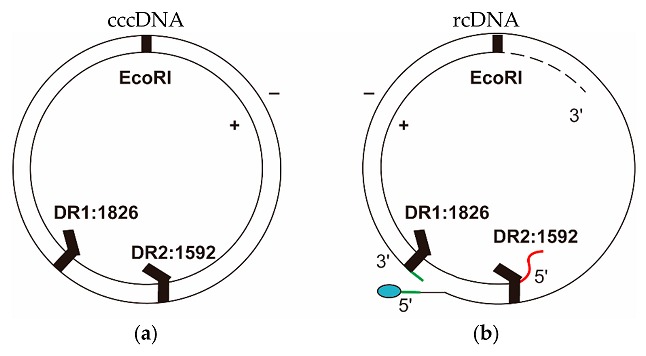
Structure of cccDNA and the HBV genomic rcDNA: (**a**) cccDNA has two complete strands: the minus-strand on the outer side and the plus-strand on the inner side. There are two direct repeats (DRs) at nucleotide (nt) 1826 and 1592 (gi: 4323196), and the position of origin is at the EcoRI site [64]; (**b**) rcDNA has a complete minus strand with a nine-base terminal redundancy (green line) and a terminal protein polymerase attached to the 5′-end (blue oval). The plus strand has a defined 5′-end with an RNA primer (red line) but a variable 3′-end (dashed line).

Compared with cccDNA, which has a pair of complete strands, the HBV genomic rcDNA has an incomplete plus strand with a variable 3′-end but a defined 5′-end near DR2 and a complete minus strand with a terminal redundancy of 9 bases at defined 5′- and 3′-ends but a gap near DR1. The schematic structures of cccDNA and rcDNA are shown in Figure 2. Differences in structure are exploited to isolate cccDNA and design specific primers for its detection [39,42,43,63].

## 3. Preparation of cccDNA Samples

**Figure 3:**
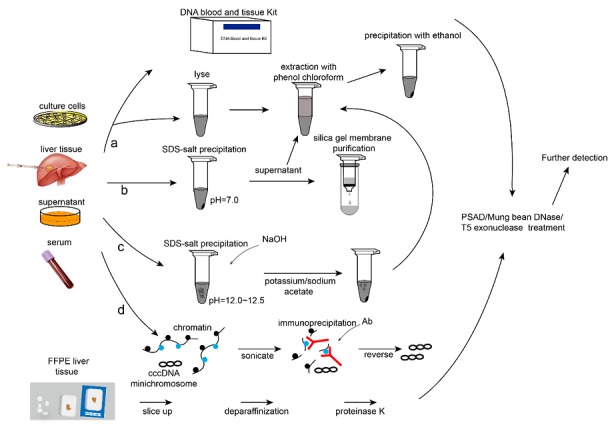
Various methods for cccDNA sample preparation. Methods (**a**–**d**) are used for cccDNA extraction and purification: Chromosomal DNA and viral DNA (protein-free DNA including cccDNA and protein-bound DNA such as intracellular HBV DNA encapsulated by HBcAg) can be purified using method (**a**), which extracts total DNA; only protein-free DNA can be isolated with methods (**b**) and (**c**). Method (**b**) is also known as the Hirt or modified Hirt procedure for rapid extraction of extrachromosomal protein-free DNA, including protein-free dslDNA, rcDNA and cccDNA; Method (**c**) is a modification of the alkaline lysis procedure for isolation of plasmid DNA. Specific protein-bound cccDNA is selectively precipitated by corresponding antibodies (Ab) in method (**d**). For in situ hybridization of cccDNA, formalin-fixed paraffin-embedded (FFPE) liver biopsy tissues are prepared differently. Nucleases [plasmid-safe adenosine triphosphate-dependent deoxyribonuclease (PSAD), mung bean DNase or T5 exonuclease] are used to degrade contaminating non-cccDNA, after which the sample is subjected to further detection procedures. SDS: sodium dodecyl sulfate.

The preparation of cccDNA samples is crucial to specifically detecting cccDNA. Because cccDNA exists in the nucleus as episomal minichromosomes in two forms (loose and condensed cccDNA minichromosomes), various isolation methods have been reported. We summarize the general outline for a variety of cccDNA sample preparations in Figure 3. Isolation of all DNA (viral DNA and chromosomal DNA), including the two forms of cccDNA, requires the lysis of the cell nucleus to release DNA and the removal of proteins. Lysis buffer containing sodium dodecyl sulfate (SDS), proteinase K or guanidine thiocyanate, 2-mercaptoethanol and 2% Sarkosy is often used for this purpose, followed by centrifugation to discard the cell debris. The supernatant is further extracted with phenol chloroform and precipitated with ethanol [20,32,33,42,45,65,66]. The QIAamp^®^ DNA blood & tissue Mini Kit (QIAGEN, Hilden, Germany) and NucleoSpin^®^ Tissue kit (Macherey-Nagel, Düren, Germany) have also been used to extract total DNA in many reports [26,30,31,35,38,39,40,41,44,67,68,69]; however, the HBV DNA species and ratios recovered from the QIAGEN and NucleoSpin^®^ Tissue kit have never been validated by Southern blot and compared to the conventional total DNA method. Because cccDNA accounts for a small proportion of total DNA, this method is not optimal for specific cccDNA detection. In 1967, Hirt proposed a method for the purification of extrachromosomal DNA that involves processing cells with SDS lysis-salt precipitation at neutral pH [70] and extraction of the supernatant by phenol chloroform. However, this method is laborious and time-consuming even with modification [36,55,57,66,71,72,73]. With the appearance of ion-exchange chromatography and silica-based resins, the Hirt procedure was modified such that cell debris and chromosomal DNA-protein complexes are discarded after SDS lysis-salt precipitation, and the supernatant is added into the pre-equilibrated column and eluted [74]. When low-molecular weight (low-MW) extrachromosomal DNA without protein covalently bound, including protein free (PF)-rcDNA, PF-dslDNA (polymerase removed) and histone-associated cccDNA, is extracted using the Hirt or modified Hirt method, further removal of non-cccDNA is necessary. To specifically purify cccDNA, which is like a plasmid, a modification of the alkaline lysis procedure for the isolation of plasmid DNA was applied as follows [53,75,76,77,78,79]: the sample was lysed in SDS-containing buffer, and NaCl was added to precipitate genomic DNA and proteins, after which the supernatant was denaturated by NaOH, neutralized by potassium acetate or sodium acetate, and finally extracted with phenol–chloroform and precipitated with ethanol. cccDNA can be extracted by the above-mentioned methods. Since the integrity of cccDNA chromatin may be maintained under certain sonication conditions, Pollicino and coworkers took advantage of chromatin immunoprecipitation (ChIP) assays using histone, histone deacetylase 1 (HDAc1), histone acetyltransferase p300 (p300) and CREB binding protein (CBP), HBcAg or HBx-specific antibodies to further study the protein profiling and modifications associated with cccDNA [14,54,80,81]. 

The methods described above are for the extraction and enrichment of cccDNA. However, in situ cccDNA analysis in formalin-fixed paraffin-embedded (FFPE) liver tissues requires different procedures that will be described in the section on cccDNA detection and quantification [21,28]. Before further detection of cccDNA, nucleases such as plasmid-safe adenosine triphosphate (ATP)-dependent deoxyribonuclease (PSAD), mung bean DNase or T5 exonuclease are often used to degrade contaminating non-cccDNA forms [20,21,28,30,31,38,39,54,63,69], while there are different studies showing that PSAD is not able to clear all rcDNA [56,67].

## 4. cccDNA Detection and Quantification

After the cccDNA sample is prepared, it is subjected to further detection procedures. Persistent efforts are being made to develop strategies for cccDNA detection. Methods that have been previously developed and applied for the detection of cccDNA are listed in Table 1.

### 4.1. Southern Blot

The Southern blot is a classical but sophisticated method for cccDNA detection that involves several processes including probe preparation, electrophoresis, transmembrane hybridization and detection [82]. To verify the presence of cccDNA, the extracted DNA needs to undergo further denaturation and/or restriction endonuclease digestion [49,57,71,76,78,82,83]. There are radioactive and non-radioactive systems for HBV probe preparation and detection: the ^32^P probe system and the digoxigenin (DIG) or biotin probe system [82,97,98,99]. The former is more sensitive and reproducible, although safety concerns limit its use. The DIG or biotin probe system is safer and has comparable sensitivity, which makes it a better alternative. However, both systems are insensitive, complicated, costly and time-consuming compared with quantitative PCR (qPCR). These limitations render the high-throughput screening of cccDNA by this method impractical. Nevertheless, Southern blotting remains a widely accepted method for the qualitation and relative quantification of cccDNA in cells in spite of the emergence of many new methods over the years [20].

**Figure 4:**
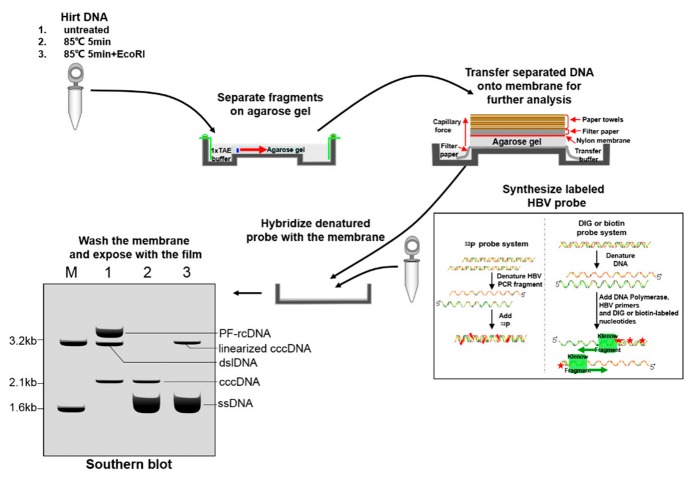
Detection of HBV cccDNA by a Southern blot assay in HepAD38. The typical pattern of protein-free Hirt DNA is shown after gel electrophoresis, transmembrane, hybridization and detection. M is an HBV DNA marker composed of 1.6 kb and 3.2 kb HBV fragments. The untreated extract (lane 1) contains all types of extrachromosomal protein-free DNA: PF-rcDNA, dslDNA and cccDNA. After 5 min 85 °C treatment before gel loading, PF-rcDNA and dslDNA turn into ssDNA and only cccDNA remains undenatured and immobile (lane 2). When it is further digested with EcoRI, cccDNA is linearized to dslDNA (lane 3).

Figure 4 illustrates the general procedure of Southern blot assay for detection of HBV cccDNA. To validate the identity of cccDNA, Hirt procedure-derived DNA samples are usually divided into three equal aliquots that are treated as follows: no treatment (lane 1), 85 °C denaturation for 5 min (lane 2) and further EcoRI digestion (lane 3). These three aliquots of DNA samples are usually analyzed by Southern blot simultaneously, thus allowing to distinguish the cccDNA from other viral DNA species by differential mobility during gel electrophoresis. A typical pattern of Hirt DNA samples from HBV-replicating HepAD38 cells upon Southern blot hybridization is schematically described in Figure 4, according the results reported in previous studies [36,82]. Typically, extrachromosomal PF-DNA, including cccDNA, PF-rcDNA and PF-dslDNA can be observed in lane 1 loaded with non-treated Hirt DNA sample. The heat treatment (85 °C for 5 min before gel loading) denatures PF-rcDNA and dslDNA into single-stranded (ss) DNA, whereas the cccDNA stays undenatured and its electrophoretic mobility remains unchanged (lane 2). When it is further digested with EcoRI after denaturation, cccDNA is linearized to genome-length dslDNA, which appears at the 3.2 kb position.

### 4.2. Real-Time qPCR

#### 4.2.1. Conventional qPCR

Conventional qPCR of cccDNA dates back to 1993 when it was developed by Kock [46], who designed a pair of cccDNA-specific primers across the gaps present on both strands of the HBV genome [46,84]. Because of the partial homology between rcDNA and cccDNA, specificity is compromised in the presence of abundant rcDNA. To reduce rcDNA contamination, TaqMan probes binding the same strand with one primer but on the other hand were devised and used in the PCR procedure [43,44]. Shao et al. introduced a chimeric sequence composed of two segments to increase specificity [42]: segment A is complementary to the HBV DNA plus strand from nt 1615 to nt 1604, and segment B is consensual to the human immunodeficiency virus (HIV) long terminal repeat (LTR) region, which differs from the HBV DNA sequence. Quantification of cccDNA includes two steps: single-strand elongation from cccDNA with the chimeric sequence, followed by real-time PCR with the aforementioned single strand as the template, one primer (P1) consensual to segment B of the chimeric sequence, the other primer (P2) upstream of nucleotides 1528 to 1547 and a probe between the primers. In 2004, Bowden introduced two sense and one antisense PCR primers to enhance the sensitivity [39]. To further increase the sensitiveness and accuracy, Singh et al. applied two hybridization fluorescence resonance energy transfer (FRET) probes in LightCycler^TM^ (Roche Diagnostic, Mannheim, Germany) real-time PCR [41] which were able to maintain a cccDNA:rcDNA specificity ratio greater than 1:10,000. Due to the inherent speed, simplicity and sensitivity of qPCR, subsequent efforts to optimize general real-time PCR conditions [11,12,13,26,32,33,34,38,54,63,69,85,86,87,88,89] (including primers, probe design and sample handling) to effectively distinguish cccDNA from other HBV DNA forms have been made without sacrificing the high amplification efficiency and sensitivity of the method.

#### 4.2.2. Competitive qPCR

The competitive qPCR assay for cccDNA detection was devised by Addison et al. in 2000 [45]. It involves two templates: a competitor template of known quantity and a target template of unknown quantity. The templates combine and compete for the same cccDNA-specific primers with comparable amplification efficiency in PCR. The lengths of PCR products for the competitor and target templates are different and can be further quantified. Based on the principles described above, when two PCR products are of equal band, as assessed by Southern transfer using a Fuji Phosphoimager system and Image Gauge software, the quantities of target and competitor templates are equal. To find the equivalence point, a series of PCRs are performed with the same unknown amount of target template and known dilutions of the competitor template. After PCR, the quantity of template increases, and sensitivity is higher than that of direct Southern blot detection. Compared with traditional quantitative PCR, this assay is more specific and accurate because of the low efficiency of cccDNA-specific primers combining with rcDNA, resulting from the special competitor template design. However, unexpected amplification signals derived from rcDNA are still possible when the amount of rcDNA is extremely high. Moreover, electrophoresis and Southern blot with ^32^P-labeled probe detection are indispensable in such an assay, which makes it difficult to be standardized.

#### 4.2.3. Semi-Nested and Nested qPCR

In 1993, Kock firstly put forward semi-nested PCR to differentiate duck hepatitis B virus (DHBV) cccDNA and rcDNA [46]. For the first PCR, P1 and P3 were used, and products were generated only when the template cccDNA was above acertain concentration, as the primer pairs were partially complementary. The generated products were further used as templates for a second PCR with P1 and another primer, P2, which is located between the P1 and P3 sequences. Mason et al. thereafter quantified HBV cccDNA with a modified primer design using the same methodology [90]. Nested qPCR was reported by Stoll-Becker [91] and Xu et al. to quantify cccDNA in peripheral blood mononuclear cells (PBMCs) and bone marrow mononuclear cells (MMNCs) in 2011 [31]. Two pairs of primers, outer and inner primers, were designed against double-strand breaks in rcDNA. Similar to semi-nested qPCR, the inner primers were located inside the outer primers. Two PCRs were implemented: the first PCR used the outer primers, and the second PCR used the inner primers, with the first PCR product as the template. To minimize the impact of non-cccDNA on accurate cccDNA detection, the authors used PBMCs and MMNCs that had been reported to support HBV replication as a template because HBV DNA can be massively washed away. This assay provides a new way to study cccDNA, but it needs to be implemented carefully to prevent contamination. In addition, two rounds of PCR have more potential risks to amplify unspecific signal like rcDNA.

#### 4.2.4. Droplet-Digital PCR

Trace amounts of cccDNA persist in the nucleus of infected cells after antiviral therapy, thus more sensitive and reliable systems are required to detect and quantify such trace cccDNA, which prompts the development of the droplet-digital PCR (ddPCR) system [20,100,101,102,103,104]. Samples are partitioned into tens of thousands of water-in-oil droplets; each droplet serves as an independent reactant for a traditional PCR. A droplet that contains the target sequence with detectable fluorescent signal is scored as a positive event, and if there is no signal in a droplet reaction, a negative event is recorded. Thus, the analysis result is a constellation of thousands of reactions reflecting a Poisson distribution. Mu et al. demonstrated that ddPCR in combination with PSAD pre-treatment and specific primers is capable of detecting one single copy of HBV cccDNA precisely, making it a sensitive and accurate method for cccDNA detection among currently available reports. However, practical value deviates from theoretical value when the template quantity is greater than 10^6^ copies. Investigation of the feasibility of ddPCR to monitor cccDNA in chronic hepatitis B patients is underway.

### 4.3. Rolling Circle Amplification qPCR

In clinical practice, formalin-fixed paraffin-embedded (FFPE) liver biopsy tissue is used for routine pathologic examination and is more readily available than freshly isolated liver biopsy tissue. However, the quantity of cccDNA in FFPE liver tissue is 100-fold lower than that in cryo-preserved liver tissue [32], such that the sensitivity of regular qPCR cannot meet pathologists’ needs. Zhong et al. combined rolling circle amplification (RCA) with real-time PCR to address this problem [30,105]. Four pairs of primers were designed for RCA with Phi29 DNA polymerase. The RCA product then served as the template for further real-time PCR. Introduction of RCA substantially improved the sensitivity and specificity of cccDNA assays and minimized the interference of integrated HBV DNA, which was overlooked in classical qPCR. However, the RCA method is very time-consuming.

### 4.4. Rolling Circle Amplification-In Situ qPCR

So far, most cccDNA quantitation methods have not been able to reveal the distribution and localization of cccDNA in liver tissue. To solve this problem, Zhong et al. further combined RCA and in situ PCR (IS-PCR) in FFPE liver tissue [28]. Tissue was cut into sections, sections were stained with hematoxylin and eosin (HE), and deparaffinization, proteinase K and PSAD digestion were performed before RCA. After RCA treatment, cccDNA-selective primers labeled with digoxigenin at the 5′-terminus and other PCR components were added to the tissue slide, and the slide was sealed, encapsulated and put into the thermal cycler for PCR. Immediately after IS-PCR, the slide was fixed, permeated, blocked, incubated with anti-digoxin alkaline phosphatase-conjugated antibody and visualized. Finally, the slide was counterstained and mounted for microscopy. Two copies of cccDNA per cell can be detected easily with this method, although the in situ PCR may result in the diffusion of amplified DNA to neighboring cells, and cross-linked histones or other cccDNA-binding proteins could hinder effective amplification of PCR [21].

### 4.5. Magnetic Capture Hybridization qPCR

For specific cccDNA quantification, selective isolation and enrichment of cccDNA is essential. In 2015, magnetic nanoparticles were first introduced in specific cccDNA capture; with this method, captured cccDNA is released by denaturation and further processed for traditional qPCR [27]. Magnetic beads were synthesized using reverse micro-emulsion method, then modified with streptavidin (SA) [106,107,108]. Selective probes targeting both sides of the rcDNA gap were designed and labeled with biotin at the 5′-terminus. The magnetic nanoparticle–SA–biotin–cccDNA probe complex was optimized and proven to hybridize specifically with cccDNA. After cccDNA in the sample solution hybridizes to the complex, the supernatant was washed away, and qPCR components were added. The cccDNA was then detached from the complex by high temperature denaturation, which was followed by conventional PCR quantification. The enrichment of cccDNA by magnetic capture hybridization (MCH) decreases the interference of rcDNA by enrichment of cccDNA and thus increases the specificity of cccDNA qPCR. This method was unable to capture all cccDNA molecules, but the difference between the detected concentration and the expected concentration was within the acceptable range. However, the materials needed for this assay make it less economical than conventional qPCR.

### 4.6. Invader Assay

The Invader assay is a non-PCR signal amplification assay for genotyping and gene expression monitoring that detects only one strand of a DNA duplex [109,110]. It was first employed by Wong et al. to quantify cccDNA in patients’ sera [40]. The method requires two oligonucleotides, the primary probe and the Invader probe, and a FRET cassette. The two oligonucleotides hybridize to the target DNA to form a partially overlapping structure that is cleaved by a cleavase enzyme to generate a 5′-flap from the primary probe. Another primary probe will cycle to the target DNA at a specific temperature, hybridize with the Invader probe and form an overlapping structure again. The released 5′-flaps increase proportionally to the concentration of the target DNA. The FRET cassette is used to react with the released 5′-flaps and generate a fluorescence signal measurable with real-time PCR machines. The differences between cccDNA and other forms of HBV DNA are utilized for the design of primary and Invader probe sequences, resulting in positive signals for cccDNA and negative signals for non-cccDNA [35,37,92]. The Invader assay provides a specific and simple method for cccDNA detection comparable with PCR, although the residual dslDNA and integrated HBV DNA can interfere with quantification, so the extracted DNA requires further treatment to eliminate interferents.

### 4.7. In Situ Hybridization

In situ hybridization (ISH) for cccDNA detection was first reported in 1998 using a DIG-labeled single-stranded probe in HBV producing HepG2 cells, but cccDNA in liver tissue was not tested [93]. Recently, a method modified from the ViewRNA assay was proposed by Zhang et al. [21]. This is a highly sensitive and specific method to visualize cccDNA with a probe set spanning the gap in rcDNA. Procedures for cccDNA ISH include sample preparation, pretreatment with protease, nuclease treatment to digest RNA or other forms of DNA, target hybridization, signal amplification and nitro blue tetrazolium (NBT)/5-bromo-4-chloro-3-indolyl-phosphate (BCIP) staining. For target hybridization, the cccDNA-specific probe set hybridizes to the target sequence, and subsequent signal amplification using branched DNA (bDNA) technology is performed on the specific hybridization of adjacent probe set oligonucleotide pairs. An 8000-fold amplification is reported by the manufacturer of the ViewRNA assay, which could meet the required sensitivity for detection of low-content nucleic acid. A series of rigorous controls were also performed in parallel to verify the specificity of the assay. Compared with visualized RCA-IS qPCR, concerns about the diffusion of amplified DNA to nearby cells or cross-contamination are not relevant to this method. Moreover, different forms of DNA and RNA can be distinguished easily, and proteins can be located by combining the method with immunohistochemistry (IHC) or immunofluorescence. 

### 4.8. Surrogate Markers of cccDNA

In addition to direct detection and quantitation, some surrogates of cccDNA were reported. Zoulim et al. found that cccDNA decline in biopsies was correlated with HBsAg decline in serum during adefovir dipivoxil therapy [12,63], suggesting that serum HBsAg quantification may represent a non-invasive surrogate marker of the intrahepatic cccDNA pool. In 2006, Zhou et al. showed that HBV precore mRNA and secretion of its translation product HBeAg in HepAD38 cells were strictly cccDNA-dependent and that the level of HBeAg in culture medium was quantitatively correlated with the amount of cccDNA in cell nuclei [36]. Hence, the cell-based HBeAg reporter assay was further studied as a convenient and cost-effective tool for high-throughput screening of drugs targeting cccDNA [29]. Cai et al. screened an in-house small-molecule library to target cccDNA in a similar cell line, HepDE19, by monitoring HBeAg in the supernatant. Two structurally-related compounds were discovered and confirmed as inhibitors of cccDNA biosynthesis for the first time [94]. 

Because the HBeAg has high homology in amino-acid sequence with the HBcAg that is cccDNA-independent in both HepDE19 and HepAD38 cells, a highly specific anti-HBV e antibody (HBeAb) remains unavailable. Therefore, direct quantification of cccDNA-dependent HBeAg suffers from possible interference from HBcAg. To overcome this problem, Guo’s group further developed the new cell line “HepBHAe82”, based on the principles of HepDE19 [25]. They introduced an in-frame human influenza hemagglutinin (HA) epitope tag into the precore domain of HBeAg without disrupting inducible HBV replication and cccDNA-dependent HA-tagged HBeAg (HA-HBeAg) expression. HA served as an intermediate that was captured by HA-antibody and used for further enzyme-linked immuno sorbent assay (ELISA) or chemiluminescent immunoassay (CLIA) assays to detect secreted HBeAg. Recent research on the correlation between Hepatitis B core-related antigen (HBcrAg) or pgRNA in patients’ sera and cccDNA levels in cells suggests that the amount of circulating HBcrAg or pgRNA may mirror the levels of transcriptionally active intrahepatic cccDNA [22,24,95]. 

With advances in technology, the production of recombined cccDNA (rcccDNA) is no longer a problem, and minicircle (MC) DNA vector-based and HBV-GLuc (*Gaussia* luciferase) cccDNA technology has been used to produce cccDNA in bacteria [23,96]. The resulting cccDNA with a separated *Gaussia* luciferase gene closely resembles its counterpart in structure and function, and it can be transfected into any type of cell to produce HBV-GLuc viruses. The *Gaussia* luciferase is expressed and secreted into the supernatant only after the intron is spliced from the pre-pgRNA, generating a functional pgRNA. Theoretically, the pgRNA in the supernatant is a surrogate of the transcriptional activity of intracellular cccDNA, consistent with the available literature. However, measurement methods for pgRNA are limited and are not applicable for high-throughput screening. The introduced *Gaussia* luciferase in the supernatant overcomes these problems and reflects the activity of bona fide cccDNA, while the insertion compromises the virus yields. Since these substituted markers are secreted into the supernatant of cultured cells or in animals’ sera, the method is non-invasive, convenient and suitable for high-throughput compound screening. However, these methods involve surrogate markers of cccDNA, and they may not reflect the entirety of information about cccDNA itself.

## 5. Conclusions and Discussion

cccDNA plays a key role in chronic hepatitis B virus infection, viral reactivation after drug withdrawal and drug resistance. Elimination of cccDNA from hepatocytes is regarded as the holy grail for achieving eradication of chronic HBV virus infection [18]. However, little is known about the biology of cccDNA due to a lack of sensitive and accurate detection methods. Monitoring of cccDNA before, during and after antiviral therapy is also essential for routine management of chronic hepatitis B patients because it is an important predictor of outcomes [11,69,111]. A variety of methods for cccDNA detection and quantification have been developed for basic research and clinical application. Most methods have been demonstrated in each article to have high sensitivity and specificity. Still, there is no universal accurate and practical method except the laborious and insensitive Southern blot, which is unsuitable for high-throughput screening targeting cccDNA. 

There are several concerns about PCR-based cccDNA detection methods for DNA recombination generated from rcDNA, and these have been recently confirmed by Wain-Hobson and coworkers [112,113]. Although samples are digested with a nuclease (PSAD) that is commonly used to specifically remove non-cccDNA before PCR quantification, there is still doubt about whether non-cccDNA is completely degraded by the nuclease digestion. Some studies demonstrated that PSAD cannot digest mature rcDNA [56,67] and that the activity of these nucleases varies depending on the amount and type of contaminating DNA. In addition to that, different primers and probes are used in the PCR reactions. With regard to Invader assays, interference from double-stranded and integrated HBV DNA is a notable deficiency, and the extraction product also needs further treatment to eliminate non-cccDNA. In situ hybridization has been developed recently, and its specificity needs further validation. Surrogate markers for cccDNA seem to be better choices because antigen quantification is easier, but some information may be missed.

A variety of sample preparation methods are used for cccDNA detection, and they have varying efficiency, which makes comparisons between studies unfeasible. The Hirt or modified Hirt extraction method to deplete protein-bound DNAs is widely used for sample preparation. However, the species and ratio of extracted protein-free DNA is different even with an identical cell line; PF-rcDNA, dslDNA and cccDNA can be extracted and detected by Southern blot, but depending on whether there is ssDNA, different studies have diverse results [49,56,57,71]. Besides, this method may miss some cccDNA covalently bound to proteins, which is also a flaw of the alkaline lysis procedure for cccDNA isolation. The whole extraction of intracellular DNA containing all forms of HBV DNA does indeed pose a challenge for further specific cccDNA detection. Guidelines or standard operation procedures need to be drawn up for cccDNA sample preparation and detection to compare different studies effectively. 

There is no doubt that cccDNA exists in HBV-infected hepatocytes, but it is controversial whether extra-hepatic tissues contain cccDNA and whether cccDNA is released into the serum. Some reports have shown that cccDNA is present in patients’ sera and that it is a marker of off-treatment virological relapse [11,26,32,34,114], whereas others have refuted the existence of cccDNA in serum and have used DNA extracted from CHB patients’ sera as a negative control in the quantification of intracellular cccDNA [39,42,63]. Do some non-liver cells, such as PBMCs, support HBV infection and formation of cccDNA? Some researchers speculate that PBMCs support only HBV replication rather than infection and that cccDNA cannot be formed in these cells [84], but others have detected cccDNA in PBMCs [38,115,116]. However, there should certainly be HBV in extra-hepatic cells since recurrence occurs after orthotopic liver transplantation (OLT). Scientists should put more effort into technological developments to visually track or label cccDNA specifically (e.g., a cccDNA fluorescent reporting system just like the replication-competent green fluorescence protein (GFP)-expressing influenza virus) [117,118], which will offer visual and quantitative information about cccDNA in each cell without a considerable impact on virus replication. This would help to solve the disputed issues and provide a powerful tool for further research on cccDNA. In addition to cccDNA quantification, scientists should also develop differential DNA denaturation PCR (3D-PCR) to analyze cccDNA modification and mutation [68,89]. In addition, methods to distinguish wild-type cccDNA from defective cccDNA are needed to effectively target functional cccDNA.

## Figures and Tables

**Table 1:** Comparison of various methods for cccDNA detection.

Methods	Advantages	Disadvantages	Dynamic Range ^1^	Limit of Detection ^1^	References
Southern blot	Reliable; reproducible.	Complicated; costly; time-consuming; insensitive; safety concerns.	More than 10 fg DNA	10 fg DNA (~2 × 10^6^ copies)	[47,48,49,55,56,57,59,65,68,71,72,76,78,81,82,83]
Conventional quantitative polymerase chain reaction (qPCR)	Simple; rapid; accurate; economical; sensitive; high-throughput.	Specificity is not absolute when rcDNA is massive.	2 × 10^3^–2 × 10^12^ copies/mL	2.7 × 10^2^ IU/mL (2 × 10^3^ copies/mL)	[11,12,13,26,32,33,34,38,39,41,42,43,44,46,47,48,54,63,68,69,84,85,86,87,88,89]
Competitive qPCR	More sensitive than Southern blot; rcDNA and cccDNA can be readily distinguished.	Specificity is not absolute when rcDNA is massive; Southern blot with ^32^P-labeled probe detection is indispensable.	2.5–60 ng DNA	2 × 10^4^ copies	[45]
Semi-nested and nested qPCR	Sensitive; specific.	May be contaminated; more risks to amplify unspecific signal.	3.0 × 10^2^–3.9 × 10^8^ copies/mL	3.0 × 10^2^ copies/mL	[31,46,90,91]
droplet-digital PCR	Super-sensitive; accurate.	Upper detection limit is restricted.	1–10^6^ copies	1 copy	[20]
Rolling circle amplification qPCR	Practical; sensitive; specific.	Time-consuming; cross-linked proteins could hinder effective amplification.	10^2^–10^10^ copies/mL	10^2^ copies/mL	[30]
Rolling circle amplification–in situ qPCR	Sensitive; visible at single-cell resolution.	Diffusion of amplified DNA to neighboring cells; cross-linked proteins could hinder effective amplification.	More than 2 copies/cell	2 copies/cell	[28]
Magnetic capture hybridization qPCR	Enrichment of cccDNA; sensitive; reproducible; specific.	Cannot capture all cccDNA; complicated; costly.	10^2^–10^6^ IU/mL	90 IU/mL	[27]
Invader assay	Specific; simple; reproducible.	Minimal interference from double-stranded and integrated HBV DNA.	10^4^–10^9^ copies/mL	50 copies (10^4^ copies/mL)	[35,37,40,92]
In situ hybridization	Specific; visible at single-cell resolution; can distinguish and locate different DNA, RNA and proteins; without diffusion of amplified products.	Complicated probe design.	More than 1 copy	1 copy under optimal conditions	[21,93]
Substituted markers ^2^ (HBsAg/HBeAg/HBcrAg/pgRNA/GLuc)	Non-invasive; convenient; cost-effective; high-throughput.	Indirect and may not reflect all information about cccDNA.	-	-	[12,22,23,24,25,29,36,63,94,95,96]

^1^ The dynamic range of detection methods reflects the maximum range among the reports. The limit of detection reflects the minimum value for the relevant methods; ^2^ The dynamic range and limit of detection are not ascertainable because the substituted markers are different; they vary due to the use of different methods to measure the corresponding markers. qPCR: Quantitative PCR; GLuc: *Gaussia* luciferase.
